# Use of Spices and Herbs to Reduce Overconsumed Dietary Components, Enhance Sensory Properties of Foods, and Improve Diet Quality

**DOI:** 10.1093/nutrit/nuaf287

**Published:** 2026-05-26

**Authors:** Kristina S Petersen

**Affiliations:** Department of Nutritional Sciences, Pennsylvania State University, University Park, PA 16802, United States

**Keywords:** spices and herbs, reformulation, saturated fat, sodium, added sugars, diet quality

## Abstract

In the United States, most individuals exceed intake recommendations for saturated fat, sodium, and added sugars, and have poor diet quality as assessed by adherence to the Dietary Guidelines for Americans. This article summarizes research funded by McCormick Science Institute to assess the potential for spices and herbs to preserve the acceptability of commonly consumed foods reformulated to be lower in saturated fat, sodium, and/or added sugars and meaningfully reduce population-level intake of these overconsumed dietary components. This proof-of-concept work involved reformulation of 10 recipes that represent leading contributors to US adult intake of saturated fat, sodium, and added sugars. Findings show that intake of the reformulated foods, instead of the original versions, by 25% to 100% of current consumers, would be expected to lower daily intake of saturated fat (-2.9% to -11.4%), sodium (-3.2% to -11.5%), and added sugars (-0.5% to -2.7%), and increase diet quality (2.1% to 6.7%). Blind taste testing showed that the overall liking ratings for 7 of the 10 reformulated foods were at parity or greater than the original foods when spices and herbs were used to enhance the flavor of the recipes. This research demonstrates that the use of spices and herbs to flavor recipes lower in overconsumed dietary components has the potential to reduce intake of saturated fat, sodium, and added sugars and is acceptable to consumers.

## INTRODUCTION

In the United States, recommendations for saturated fat, sodium, and added sugars are exceeded by most individuals aged 2 years and older.^1^ Furthermore, diet quality as assessed by adherence to the Dietary Guidelines for Americans, is suboptimal across all life stages.^1^ Therefore, approaches are needed to reduce intake of saturated fat, sodium, and added sugars, and increase diet quality.

The 2020–2025 Dietary Guidelines for Americans recommend using spices and herbs to flavor foods lower in saturated fat, sodium, and added sugars.^1^ This recommendation is based on research showing that the addition of spices and herbs to foods lower in added sugars and saturated fat preserves liking scores.^2^^,^^3^ Furthermore, clinical research has demonstrated that the use of spices and herbs assists in the maintenance of a low-sodium diet.^4^ However, the potential population-level impact of using spices and herbs to improve the flavor profile of foods lower in overconsumed dietary components (ie, saturated fat, sodium, and added sugars) remains unclear.

To further understand the potential for spices and herbs to preserve the acceptability of commonly consumed foods lower in overconsumed dietary components, the McCormick Science Institute funded proof-of-concept research to answer the following research question: Does adding spices and herbs to commonly consumed foods reformulated to be lower in saturated fat, sodium, and/or added sugars meaningfully improve population-level intake of these overconsumed dietary components and is this approach acceptable to consumers? This program of work included 4 components: (1) identification of 10 leading contributors to US adult intake of saturated fat, sodium, added sugars, and energy using 2015–2018 National Health and Nutrition Examination Survey (NHANES) data that could be feasibly reformulated; (2) reformulation of the 10 leading contributors to intake of overconsumed dietary components to reduce saturated fat, sodium, and/or added sugars with the addition of spices and herbs to maintain acceptability; (3) modeling of the potential influence of consumer adoption of the reformulated recipes on total daily intake of saturated fat, sodium, and added sugars, and diet quality using NHANES 2015–2018; and (4) evaluation of the consumer acceptability of the reformulated recipes by blind taste testing. The results of this work were published in the *Journal of the Academy of Nutrition and Dietetics* in January 2024 and the article was featured on the cover.^5^ The present article briefly reviews this research and the key findings.

## IDENTIFICATION AND REFORMULATION OF LEADING CONTRIBUTORS TO INTAKE OF OVERCONSUMED DIETARY COMPONENTS

Using NHANES 2015–2018, the 10 leading contributors to adult intake of saturated fat, sodium, added sugars, and/or energy, which were the focus of this research, were as follows: (1) cheese pizza, (2) taco meat, (3) pasta with meat sauce, (4) macaroni and cheese, (5) brownie, (6) apple pie, (7) chili, (8) meatloaf, (9) chicken pot pie, and (10) cream sauce for chicken. These recipes were modified by culinary professionals to reduce the amount of saturated fat, sodium, and/or added sugars while maintaining the functionality and culinary aspects. Once a nutritionally improved version (ie, lower in saturated fat, sodium, and/or added sugars) of each recipe was created, another version was created that included spices and herbs. The nutritionally improved, flavor-enhanced version had the same nutritional profile as the nutritionally improved version and included spices and herbs to compensate for the flavor losses associated with the reduction in saturated fat, sodium, and/or added sugars. Across the 10 recipes, reformulation to reduce overconsumed dietary components resulted in meaningful median reductions in saturated fat (–37%) and sodium (–40%). Only 5 recipes contained added sugars, and across these recipes, the median reduction was 58%.

## MODELING THE POTENTIAL IMPACT OF THE REFORMULATION ON INTAKE OF OVERCONSUMED DIETARY COMPONENTS AND DIET QUALITY

To determine the potential population-level impact of this reformulation approach, we modeled the difference in intake of saturated fat, sodium, and added sugars under 4 different consumer adoption scenarios using NHANES 2015–2018. This included modeling intake of the reformulated version of the recipe by 25%, 50%, 75%, and 100% of current consumers compared with intake of the original version of the recipe (**Table 1**). It was estimated that daily saturated fat intake would be reduced by 2.87% (95% CI, –3.99%, –1.75%) with 25% consumer adoption to 11.40% (95% CI, –12.80%, –10.01%) with 100% consumer adoption. Similarly for sodium, intake of the reformulated recipes, instead of the original recipes, would be expected to lower sodium intake by 3.16% (95% CI, –4.63%, –1.70%) with 25% consumer adoption to 11.46% (95% CI, –13.63%, –9.29%) with 100% consumer adoption. Small reductions in estimated added sugar intake were observed across the range of modeled consumer adoption (25% adoption: -0.51%; 95% CI, –0.73%, –0.29%; 100% adoption: –2.66%; 95% CI, –3.70%, –1.61%). The smaller estimated impact of the recipe reformulation on added sugar intake is because only 5 recipes contained added sugars. In summary, the modeling showed meaningful reductions in estimated daily intake of saturated fat and sodium with this approach, demonstrating that this strategy, if widely implemented and adopted, may assist with reducing the intake of these overconsumed dietary components.

**Table 1.** Estimated Difference in Intake of Saturated Fat, Added Sugars, and Sodium with Consumer Intake of the 10 Reformulated Foods Based on the National Health and Nutrition Examination Survey (NHANES) 2015–2018

Dietary component	No modification, mean (95% CI)	Estimates based on the percentage of consumers using the modified recipes, mean % change (95% CI)			
		25% Use	50% Use	75% Use	100% Use
Saturated fat (g/d)	33.09 (31.31, 34.87)	−2.87 (-3.99, -1.75)	−5.21 (-6.20, -4.22)	−8.32 (-9.73, -6.92)	−11.40 (-12.80, -10.01)
Added sugar (tsp eq/d)	18.53 (16.34, 20.72)	−0.51 (-0.73, -0.29)	−1.05 (-1.37, -0.73)	−2.16 (-3.10, -1.22)	−2.66 (-3.70, -1.61)
Sodium (mg/d)	3798 (3570, 4027)	−3.16 (-4.63, -1.70)	−5.41 (-6.75, -4.07)	−8.18 (-9.92, -6.43)	−11.46 (-13.63, -9.29)

Abbreviation: tsp eq, teaspoon equivalent.

**Figure 1.**
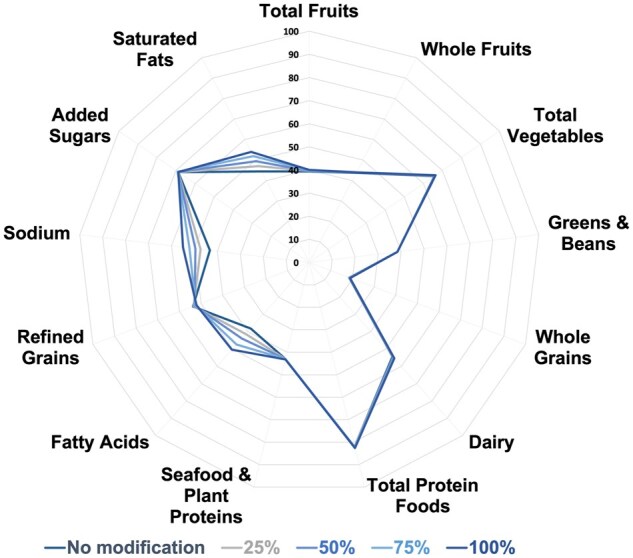
Radar Plot of the Estimated Improvement in the HEI-2020 With Consumer Intake of the 10 Reformulated Foods Based on NHANES 2015–2018 Abbreviations: HEI-2020, Healthy Eating Index–2020; NHANES, National Health and Nutrition Examination Survey.

The potential impact of this reformulation approach on diet quality, assessed by the Healthy Eating Index–2020 (HEI-2020), was also estimated. The HEI-2020 assesses adherence to the Dietary Guidelines for Americans and includes 13 components, 4 of which relate to the intake of saturated fat, added sugars, and sodium. It was estimated that, with 25% to 100% consumer adoption of the reformulated recipes, the HEI-2020 would be increased by 2.1% (95% CI, 1.2%–3.0%) to 6.7% (95% CI, 5.9%–7.6%) (**Figure 1**). This increase is driven by estimated improvements in the sodium component of 8.5% (95% CI, 3.75%–13.25%) to 21.3% (95% CI, 17.2%–25.3%) with 25% to 100% consumer adoption and improvements in the fatty acid component of 7.8% (95% CI, 4.3%–11.4%) to 24.4% (95% CI, 19.2%–29.7%). Improvements were also observed for the saturated fat component of 5.5% (95% CI, 3.0%–8.1%) to 17.6% (95% CI, 14.9%–20.4%) with 25% to 100% consumer adoption. The added sugar component was minimally improved, which is because only 5 of the recipes contained added sugars. In summary, this reformulation approach would be expected to meaningfully increase diet quality by improving intake of saturated fat and sodium.

## CONSUMER ACCEPTABILITY OF THE REFORMULATED RECIPES

Blind taste testing was conducted to assess the consumer acceptability of the nutritionally improved, flavor-enhanced recipe and the nutritionally improved recipe compared with the original recipe. Between 85 and 107 participants taste-tested each of the 10 recipes in a blinded manner. For 7 out of the 10 recipes, overall liking of the nutritionally improved, flavor-enhanced recipe was at parity or greater than the original version of the recipe. For 2 of these 7 recipes (brownie and chicken in cream sauce), the nutritionally improved, flavor-enhanced recipe was liked more than the original recipe, and for 3 recipes (chili, pasta with meat sauce, and taco meat) the liking ratings were similar for the nutritionally improved, flavor-enhanced recipe and the nutritionally improved recipe compared with the original recipe. Last, for 2 of the 7 recipes (meatloaf and apple pie), the liking ratings for the nutritionally improved recipe were significantly lower than for the original recipe, whereas the liking ratings for the nutritionally improved, flavor-enhanced recipe were not different from the original recipe. This finding suggests that the addition of spices and herbs restored the flavor profile to achieve liking parity with the original version of the recipe. For the remaining 3 recipes (cheese pizza, macaroni and cheese, and chicken pot pie), liking ratings of the nutritionally improved, flavor-enhanced recipe were lower than the original version of the recipe. These 3 recipes are higher in fat from cheese and other full-fat dairy products, and, likely, the addition of spices and herbs did not improve the acceptability of these higher-fat foods because they are liked for their textural attributes, specifically creaminess, which spices and herbs do not contribute. Collectively, these findings suggest that modification of recipes to be lower in saturated fat, sodium, and added sugars with the addition of spices and herbs to improve the flavor profile is a viable strategy to increase consumer acceptance.

## CONCLUSION

This proof-of-concept research suggests that using spices and herbs to flavor recipes lower in saturated fat, sodium, and/or added sugars is generally acceptable to consumers. If widely adopted, this approach is likely to meaningfully reduce intake of overconsumed dietary components and improve diet quality, which would be expected to have a positive influence on diet-related chronic disease. For widespread adoption, further research is needed to determine the feasibility of this approach for commercially produced foods, given the widespread consumption in the United States.

## References

[1] US Department of Agriculture; US Department of Health and Human Services. Dietary Guidelines for Americans, 2020-2025. 9th edition. Published 2020. Accessed January 1, 2021. www.dietaryguidelines.gov

[2] Peters JC , Marker R, Pan Z, Breen JA, Hill JO. The influence of adding spices to reduced sugar foods on overall liking. J Food Sci. 2018;83:814-821. 29476623 10.1111/1750-3841.14069 PMC5873279

[3] Polsky S , Beck J, Stark RA, Pan Z, Hill JO, Peters JC. The influence of herbs, spices, and regular sausage and chicken consumption on liking of reduced fat breakfast and lunch items. J Food Sci. 2014;79:S2117-S2126. 25219391 10.1111/1750-3841.12643 PMC4197100

[4] Anderson CAM , Cobb LK, Miller IER, et al Effects of a behavioral intervention that emphasizes spices and herbs on adherence to recommended sodium intake: results of the SPICE randomized clinical trial. Am J Clin Nutr. 2015;102:671-679. 26269371 10.3945/ajcn.114.100750 PMC4548171

[5] Petersen KS , Fulgoni VL, Hopfer H, Hayes JE, Gooding R, Kris-Etherton P. Using herbs/spices to enhance the flavor of commonly consumed foods reformulated to be lower in overconsumed dietary components is an acceptable strategy and has the potential to lower intake of saturated fat and sodium: a national health and nutrition examination survey analysis and blind tasting. J Acad Nutr Diet. 2024;124:15-27, e1. 37532099 10.1016/j.jand.2023.07.025

